# Transcatheter Closing Atrial Septal Defect in a Child With Hereditary Spherocytosis

**DOI:** 10.3389/fped.2019.00506

**Published:** 2019-12-17

**Authors:** Zhixian Ji, Na Liu, Zhanhui Du, Gang Luo, Zhen Bing, Quansheng Xing, Silin Pan

**Affiliations:** Heart Center, Qingdao Women and Children's Hospital, Qingdao University, Qingdao, China

**Keywords:** atrial septal defect, hereditary spherocytosis, percutaneous transcatheter closure, cardiopulmonary bypass, hemolysis

## Abstract

A 3-year-old girl was admitted to our hospital for the correction of atrial septal defect (ASD). Open heart operation with cardiopulmonary bypass is dangerous because the patient also had hereditary spherocytosis, which put her at risk for hemolytic anemia. Therefore, percutaneous transcatheter closure for ASD was chosen and performed successfully, which avoided the erythrocyte damage caused by cardiopulmonary bypass. This is the first time such a case has been reported, and we present an alternative approach for ASD with hereditary spherocytosis.

## Introduction

Hereditary spherocytosis (HS) is a genetic blood disease characterized by hemolysis, anemia, jaundice, and splenomegaly (1). Atrial septal defect (ASD) is one of most common congenital heart defects (2). However, concomitant ASD and HS is rare. Patient with HS undergoing traditional open heart surgery has a high risk of perioperative hemolysis and secondary renal dysfunction because of the deleterious effects of cardiopulmonary bypass (CPB) (3).

At present, transcatheter closure of ASD has been accepted worldwide as an alternative to surgical closure due to the ease of interventional therapy and the often increased perioperative risk from surgery (4, 5). There is no published report about children with HS undergoing interventional closure for structural heart disease. Here, we reported a case of a 3-year-old girl manifested with ASD and HS undergoing transcatheter ASD closure.

## Case Report

The patient was brought to the heart center of our hospital after a diagnosis of ASD and HS. The patient had been suffering from repeated attacks of hemolytic anemia since she was born. She was intermittently treated in the local hospitals for 2 years with the medicine “Yinzhihuang” (a traditional Chinese Medicine), L-carnitine, Vitamin B12, etc., and her hemoglobin level was maintained at 90 g/L with normal liver function. The patient presented to a local hospital 2 years ago because of the upper respiratory tract infection, and was found to have a heart murmur during the physical examination. The followed echocardiographic examination revealed an ASD, and the patient was suggested to go to check-up regularly. One month ago, the patient was sent to the local hospital for anemia and was diagnosed with HS based on the laboratory findings of the patient's blood smear and the osmotic fragility test. Then, the patient was introduced to our hospital for further treatments.

**Figure 1:**
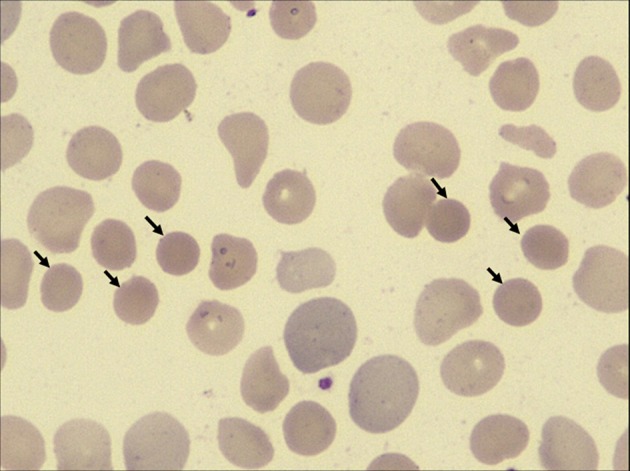
High-power view (×100 magnification) of peripheral blood smears from the patient. Spherocytes are indicated by black arrows.

On admission, the patient was anemic and icteric. She weighed 14.5 kg. Physical examination revealed a systolic ejection murmur at the left second intercostal space and hepatosplenomegaly. Laboratory findings showed a hemoglobin level of 82 g/L, and red blood cell (RBC) count of 3.26 × 10^12^/L. The total serum bilirubin was 89.4 μM, direct bilirubin was 11.8 μM, and indirect bilirubin was 77.6 μM. Elevations of the liver enzymes were not found. A peripheral blood smear revealed RBC size disparity and the presence of spherocytes (12%, Figure 1). RBC osmotic fragility was increased (hemolysis began at 0.55% NaCl and was complete at 0.50% NaCl). Additionally, a bone marrow examination was done, which showed erythroblastic hyperplasia dominated with rubricyte and metarubricyte. Echocardiography showed a left to right shunt, enlargement of right atrium and right ventricle, dilatation of main pulmonary artery, and fossa ovalis ASDs.

Concomitant ASD and HS is rare. Surgical intervention for ASD using CPB might exacerbate hemolysis and subsequent renal dysfunction because of the deleterious effects of CPB. Therefore, we consulted the hematologist and cardiac surgeons, and got a unanimous decision to perform transcatheter closure of ASD for the patient. Her parents were informed of the purpose and risk of the treatment, and written informed consent was obtained before intervention operation.

**Figure 2:**
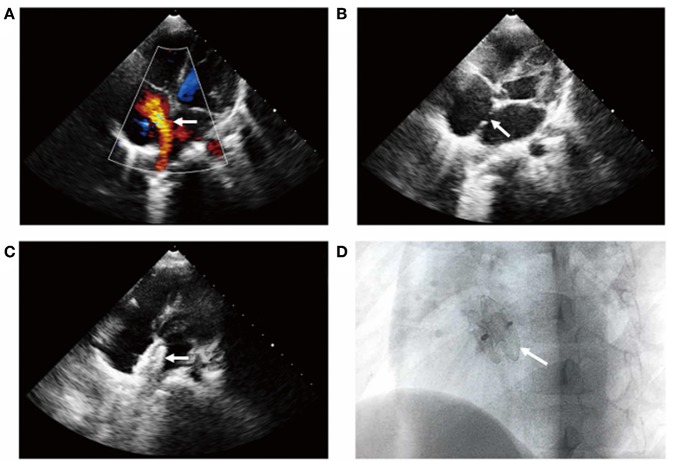
Transcatheter closure of ASD shown by transthoracic color Doppler echocardiography. The apical four-chamber section showed **(A)** left to right shunt at the level of atrial septum, **(B)** the continuous interruption of the atrial septum with a loss of about 14 mm, and **(C)** the atrial septal occluder was well-positioned without residual shunt (see arrow). **(D)** X-ray examination of left anterior oblique position after interventional closure showed that the occluder was fixed and the shape was normal (see arrow).

Percutaneous transcatheter closure of ASD was performed under general anesthesia. Echocardiography showed that the diameter of ASD was 14 × 10 mm (Figures 2A,B). The whole process of cardiac catheterization was smooth, and a 16-mm ASD occluder was placed through 8F delivery sheath (Figures 2C,D). From the first day of operation, sodium bicarbonate (2 ml/kg) was given daily to alkaline blood for 3 days and aspirin (3 mg/kg/day) was taken orally for 6 months. Multiple retests of blood routine and urine analysis had been undertaken after closure, and the results showed no decrease in hemoglobin and the results of urine analysis were normal. Echocardiography showed that the location of occluder was fixed and there was no pericardial effusion. Gradually, the patient recovered and was discharged from the hospital. One-month follow-up result showed that the level of hemoglobin remained stable and no hemoglobinuria occurred. Echocardiography showed no residual ASD and a normal state of heart function.

## Discussion

HS is a genetic, frequently familial hemolytic blood disease (1). One of the major concerns for children with HS undergoing open-heart surgery for congenital heart disease is accentuation of the risk of perioperative hemolysis. Several patients with HS undergoing open-heart surgery under CPB have been reported so far (3, 6–10). Based on previous reports, cardiac surgery has been successfully performed in all the patients. However, the potential worsening concern from CPB in HS and trauma from open-heart surgery is a big problem. A variety of approaches have been proposed for HS patients to avoid hemolysis during surgery, such as preemptive splenectomy (7). Although splenectomy is an effective treatment for reducing hemolysis, it has a high risk of infection in children under 2 years of age (10, 11). Because our patient was only 3 years old, it was not recommended to undergo splenectomy before cardiac surgery in order to maintain her immune function.

In the last 20 years, ASD transcatheter occlusion technique has been accepted worldwide as an alternative to surgery for its major advantages, such as the short hospital stay as well as evading thoracotomy, CPB, intensive care, and long scars (4, 5). Though there is also a risk of hemolysis after transcatheter closure, most scholars believe that there will be no damage to RBCs due to the hemodynamic characteristics of ASD (3). The pressure gradient between the left atrium and right atrium is small, which usually does not produce high-speed blood flow and high shear force, even if there is a small residual shunt after closure. According to the conclusion discussed by our heart center experts, the first choice for the patient to close ASD was interventional therapy by cardiac catheterization. The most important thing was to choose the appropriate occluder to avoid the use of large ASD occluder (12). The measures for preventing hemolysis and corresponding treatment were formulated in detail, and sodium bicarbonate and rehydration were used for the treatment of hydrated alkali on the day after catheterization. The level of hemoglobin and the state of heart function were observed closely. If there was severe anemia, hemolysis, and deterioration of cardiac function, aspirin could be discontinued. High dose of steroid could be used to stabilize cell membrane. If necessary, surgical removal of occluder and even splenectomy would be performed (13). The hemoglobin level of the patient was maintained at about 90 g/L, and the hematocrit was maintained at about 30%. No obvious extravascular hemolysis occurred. Additionally, there have been reported cases of HS that could lead to congestive heart failure (14). Hence, the patient still need close follow-up of the degree of hemolytic anemia and the station of cardiac function.

In conclusion, an extremely rare condition of having both ASD and HS in a same patient and successful therapeutic interventions by cardiac catheterization has been reported.

## Data Availability Statement

The raw data used to support the findings of this study are available from the corresponding author upon request.

## Ethics Statement

Written informed consent was obtained from patient's parents for publication of this case report and any potentially identifying information.

## Author Contributions

ZJ and NL drafted the initial manuscript. ZD, GL, and ZB collected the samples, clinical data, and analyzed the data. QX and SP conceived and designed the study, and revised the manuscript.

### Conflict of Interest

The authors declare that the research was conducted in the absence of any commercial or financial relationships that could be construed as a potential conflict of interest.
